# Time trajectories and within-subject correlations of matrix metalloproteinases 3, 8, 9, 10, 12, and 13 serum levels and their ability to predict mortality in polytraumatized patients: a pilot study

**DOI:** 10.1186/s40001-024-01775-x

**Published:** 2024-04-10

**Authors:** Lukas L. Negrin, Greta L. Carlin, Robin Ristl, Stefan Hajdu

**Affiliations:** 1 University Department of Orthopedics and Trauma Surgery, Medical University of Vienna, Waehringer Guertel 18-20, 1090 Vienna, Austria; 2 University Department of Obstetrics and Gynecology, Medical University of Vienna, Waehringer Guertel 18-20, 1090 Vienna, Austria; 3 Center for Medical Statistics, Informatics and Intelligent Systems, Medical University of Vienna, Waehringer Guertel 18-20, 1090 Vienna, Austria

**Keywords:** Matrix metalloproteinase, Biomarker, Time trajectory, Polytrauma, Multiple injured, Mortality, Autocorrelation, Cross-correlation

## Abstract

**Background:**

Managing polytrauma victims poses a significant challenge to clinicians since applying the same therapy to patients with similar injury patterns may result in different outcomes. Using serum biomarkers hopefully allows for treating each multiple injured in the best possible individual way. Since matrix metalloproteinases (MMPs) play pivotal roles in various physiological processes, they might be a reliable tool in polytrauma care.

**Methods:**

We evaluated 24 blunt polytrauma survivors and 12 fatalities (mean age, 44.2 years, mean ISS, 45) who were directly admitted to our Level I trauma center and stayed at the intensive care unit for at least one night. We determined their MMP3, MMP8, MMP9, MMP10, MMP12, and MMP13 serum levels at admission (day 0) and on days 1, 3, 5, 7, and 10.

**Results:**

Median MMP8, MMP9, and MMP12 levels immediately rose after the polytrauma occurred; however, they significantly decreased from admission to day 1 and significantly increased from day 1 to day 10, showing similar time trajectories and (very) strong correlations between each two of the three enzyme levels assessed at the same measurement point. For a two-day lag, autocorrelations were significant for MMP8 (− 0.512) and MMP9 (− 0.302) and for cross-correlations between MMP8 and MMP9 (− 0.439), MMP8 and MMP12 (− 0.416), and MMP9 and MMP12 (− 0.307). Moreover, median MMP3, MMP10, and MMP13 levels significantly increased from admission to day 3 and significantly decreased from day 3 to day 10, showing similar time trajectories and an (almost) strong association between every 2 levels until day 7. Significant cross-correlations were detected between MMP3 and MMP10 (0.414) and MMP13 and MMP10 (0.362). Finally, the MMP10 day 0 level was identified as a predictor for in-hospital mortality. Any increase of the MMP10 level by 200 pg/mL decreased the odds of dying by 28.5%.

**Conclusions:**

The time trajectories of the highly varying individual MMP levels elucidate the involvement of these enzymes in the endogenous defense response following polytrauma. Similar time courses of MMP levels might indicate similar injury causes, whereas lead–lag effects reveal causative relations between several enzyme pairs. Finally, MMP10 abundantly released into circulation after polytrauma might have a protective effect against dying.

## Background

Matrix metalloproteinases (MMPs) are a large family of calcium-dependent zinc-containing endopeptidases [1, 2]. There are 28 types of MMPs in vertebrates; at least 23 are expressed in human tissue [3]. These ubiquitarian enzymes catalyze the cleavage of peptide bonds within a protein [4]. MMPs contribute to the homeostasis of many tissues and are involved in tissue remodeling during various physiological processes such as angiogenesis, embryogenesis, morphogenesis, and wound repair [5, 6]. Their primary role is the degradation of extracellular matrix (ECM) proteins, glycoproteins, membrane receptors, cytokines, and growth factors [5]. Moreover, they have been connected to pathological conditions such as myocardial infarction, fibrotic disorders, osteoarthritis, and cancer [7], contributing to tumor growth, metastasis, and invasion [8]. MMPs share common functional domains and activation mechanisms. Based on their substrates or similar structural parts, they are subdivided into collagenases, gelatinases, stromelysins, matrilysins, membrane-type MMPs, and other MMPs [7]. MMP3 and MMP10 are stromelysins. MMP3 is produced by fibroblasts and platelets, and MMP10 by keratinocytes, macrophages, and epithelium [5]. MMP8 and MMP13 are collagenases. MMP8 is released by chondrocytes, endothelial cells, and macrophages, and MMP13 by connective tissue, smooth muscle, epithelial, and neuronal cells [9]. The gelatinase MMP9 is expressed by neutrophils, macrophages, polymorphonuclear leucocytes, osteoblasts, fibroblasts, granulocytes, keratinocytes, epithelial, dendritic, and T-cells [5]. Finally, MMP12 belongs to the group of “other MMPs”. It is secreted by chondrocytes, macrophages, osteoblasts, and the placenta [10].

MMPs are synthesized and normally secreted as inactive zymogens with a propeptide domain [5] that interacts with the zinc-ion bound to the catalytic site [11]. Proteolytic cleavage of this domain results in the exposure of the active site, leading to the transition of the zymogen into the active enzyme [12]. The regulation of the proteolytic activity of MMPs is mainly controlled by four endogenous tissue inhibitors of metalloproteinases (TIMPs) that bind tightly to the MMP active site [13]. Changes in actual MMP activity thus depend on the balance between the production and activation of MMPs and the local levels of TIMPs [14].

MMPs play pivotal roles in various physiological processes, primarily through their effects on remodeling the extracellular matrix, and are associated with several pathophysiological processes [15]. They affect the immune system by modulating the differentiation and activity of immune cells and recruiting macrophages and neutrophils [16]. Additionally, they are involved in catabolic processes [17, 18]. MMPs are of interest for new diagnostic and prognostic tools for the clinical management of vascular disease, cancer progression and metastasis, neurodegenerative or bone disorders, cardiovascular disease, diabetes, or sepsis [19]. However, our literature search only identified a few studies focusing on MMPs and trauma. They revealed the involvement of some MMPs in the pathophysiology of brain injury [20–23], spinal cord injury [24–27], and acute respiratory distress syndrome [28], as well as higher serum MMP9 levels in critically ill patients who did not survive [29]. Brumann et al. showed lower MMP9 serum levels within the first posttraumatic hours in patients with severe polytrauma (Injury Severity Score (ISS) > 33) compared to those with an ISS of 16–33. Moreover, MMP9 serum levels were lower in fatalities than in 30-day survivors within the first 24 h after the traumatic event [30]. Braunstein et al. found significantly higher MMP9 serum levels in polytraumatized patients with concomitant traumatic brain injury compared to those without intracranial damage six, 12, and 24 h after the injuries occurred [31].

Given these findings, we hypothesized that using serum MMP levels might be of clinical value in polytrauma management. Already at the injury site, we expected a more or less pronounced upregulation of MMP levels depending on patient characteristics and injury pattern, potentially providing curve progressions that might show approaches for treating each multiple injured in the best individual possible way. As the technology required for quantitatively determining serum levels of biomarker candidates must already be available, we had MMPs 1, 2, 3, 7, 8, 9, 10, 12, and 13 to select from. Based on the results obtained by analyzing them, we decided in retrospect to focus solely on MMPs 3, 8, 9, 10, 12, and 13 in this paper and present MMPs 1, 2, and 7 in a separate publication.

The objectives of our study were (1) to assess the time trajectories of the MMPs 3, 8, 9, 10, 12, and 13 serum levels within ten days, (2) to highlight similarities in their temporal courses, (3) to search for any associations between two MMP serum levels, and (4) to identify MMP serum levels assessed at admission as predictive biomarker candidates for mortality.

## Patients and methods

### Patients

All patients with (1) a minimum patient age of 18 years, (2) who suffered at least two blunt injuries resulting in an ISS ≥ 16, (3) who were directly admitted to the resuscitation room of our Level I trauma center from January 1 to December 31, 2019, and (4) who stayed at least one night in the intensive care unit were included in our pilot study. Patients with chronic inflammatory lung diseases or malignancies were excluded. Ten healthy adults responding to our volunteer call were combined into the control group.

### Enzyme assessment

Blood was taken from each polytraumatized patient during the initial examination at admission (day 0) and throughout hospitalization on days 1, 3, 5, 7, and 10 during the routine blood withdrawal using one separation gel tube (Vacuette R^©^ 8 mL; Greiner Bio-One International) every time. Immediately after sampling, blood was centrifuged at 3000×g for 15 min at room temperature to gain the serum, which was then isolated and stored at −80 °C until assayed. For the simultaneous quantitative determination of MMPs 1, 2, 3, 7, 8, 9, 10, 12, and 13 and TIMPs 1, 2, 3, and 4 in the serum of polytraumatized patients, we used R&D Systems^®^ “Human Magnetic Luminex^®^ Performance Assay MMP Base Kit LMPM000” and “Human TIMP Multiplex Kit LKT003”. After analyzing the similarities and associations between the assessed serum levels, we separated the 13 evaluated proteins into two groups, presenting the results in two papers due to the large amount of information.

The patients were informed about blood sampling at the earliest time point possible. If written consent was not provided, no further blood samples were taken, and the previously sampled material was destroyed if the patient requested. Only one blood sample was drawn from each participant of the control group.

### Statistical analysis

Statistical analysis was performed using the software R 3.5. and IBM SPSS Statistics 29. Demographic data are presented by mean and standard deviation, and enzyme levels are shown by median and range. Qualitative data are characterized by frequency and percentage. We used Mann–Whitney-*U*-tests for comparisons between independent groups and the Wilcoxon signed rank tests to compare enzyme levels within a patient between time points. To reveal associations between enzyme levels at the same measurement points, we calculated Spearman’s correlation coefficients. We used the correlation coefficients with repeated measurements to analyze the common intra-individual association for paired repeated measures, according to Bland and Altman [32]. To assess whether enzyme levels may predict survival, we conducted univariable binary logistic regression analyses for in-hospital mortality, with every enzyme level measured at admission as the predictor. Odds ratios (OR) are presented with 95% confidence intervals (CI). If relevant, the receiver operating characteristic (ROC) curve was plotted for graphical analysis, and the corresponding value of the area under the curve (AUC) was determined. The maximum sum of sensitivity and specificity defined the cutoff level.

## Results

### Clinical course

Thirty-six consecutive patients (23 males and 13 females) formed our study group, with an in-hospital mortality rate of 33.3%. Four patients died on day 1, three on day 4, and one on days 2, 3, 7, 11, and 42, respectively. Survivors spent an average of 37 ± 23 days in the hospital. Mechanisms of injury included pedestrian hits by vehicles (5 patients), falls from height (6 patients), traffic accidents (12 patients), being hit by an oncoming subway (1 patient), being hit by a fallen tree branch (1 patient), committed (3 patients) or attempted (4 patients) suicide by jumping, and attempted suicide by throwing themselves in front of a train (4 patients). Overall baseline characteristics are presented in Table 1.

**Table 1:** Baseline characteristics of the study group. Displayed values are mean ± standard deviation or median [minimum–maximum] or absolute (relative) frequencies

Age (years)	44.2 ± 22.1
ISS	45 ± 16
AIS_Head_ ≥ 3 (*n*)	20 (55.6%)
AIS_Face_ ≥ 3 (*n*)	3 (8.3%)
AIS_Thorax_ ≥ 3 (*n*)	29 (80.6%)
AIS_Abdomen_ ≥ 3 (*n*)	16 (44.4%)
AIS_Extremitis_ ≥ 3 (*n*)	25 (69.4%)
AIS_External_ ≥ 3 (*n*)	0 (0%)
Complications	
Sepsis (*n*)	3 (8.3%)
ARDS (*n*)	8 (22.2%)
Pneumonia (*n*)	10 (27.8%)
Acute kidney injury (*n*)	5 (13.9%)
Hemofiltration (*n*)	3 (8.3%)
Urinary tract infiltration (*n*)	3 (8.3%)
Pancreatitis (*n*)	2 (5.6%)
Clostridium difficile infection (*n*)	1 (2.8%)
Enzyme levels day 0 (pg/mL)	
MMP3	9594 [3068 − 35770]
MMP8	29,133 [5323 − 788304]
MMP9	1,360,006 [287278 − 4698005]
MMP10	1109 [392 − 2570]
MMP12	3852 [1609 − 6418]
MMP13	1943 [711 − 3413]
Reference levels (pg/mL)	
MMP3	7213 [3510 − 21172]
MMP8	7161 [3628 − 49119]
MMP9	454,554 [389036 − 1351769]
MMP10	797 [419 − 2121]
MMP12	2172 [1930 − 3702]
MMP13	1926 [1040 − 3728]

*ISS* Injury severity score, *AIS* Abbreviated injury scale, *ARDS* Acute respiratory distress syndrome

### Trend curves

**Fig. 1:**
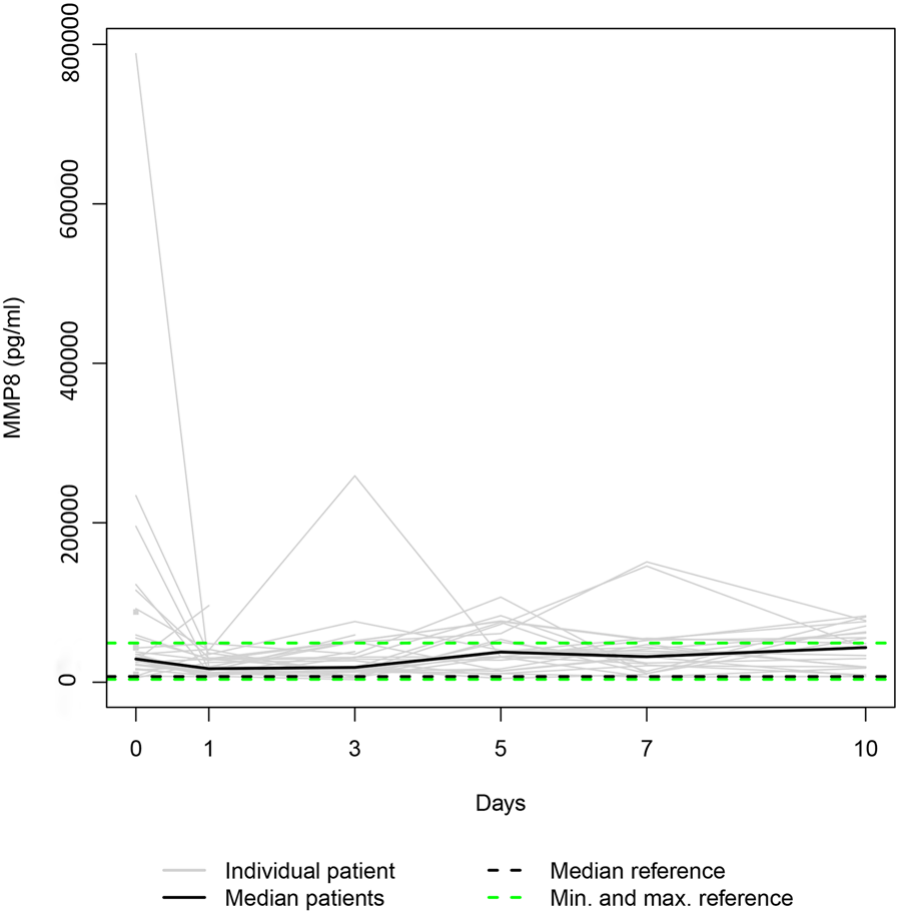
Individual MMP8 serum levels (gray lines) and the median MMP8 serum level (bold black line) in the study group. Median (dashed bold black line) and minimum and maximum (dashed green lines) MMP8 serum levels in the healthy volunteers. The gray squares on the plots represent the patients with measurements only at admission

**Fig. 2:**
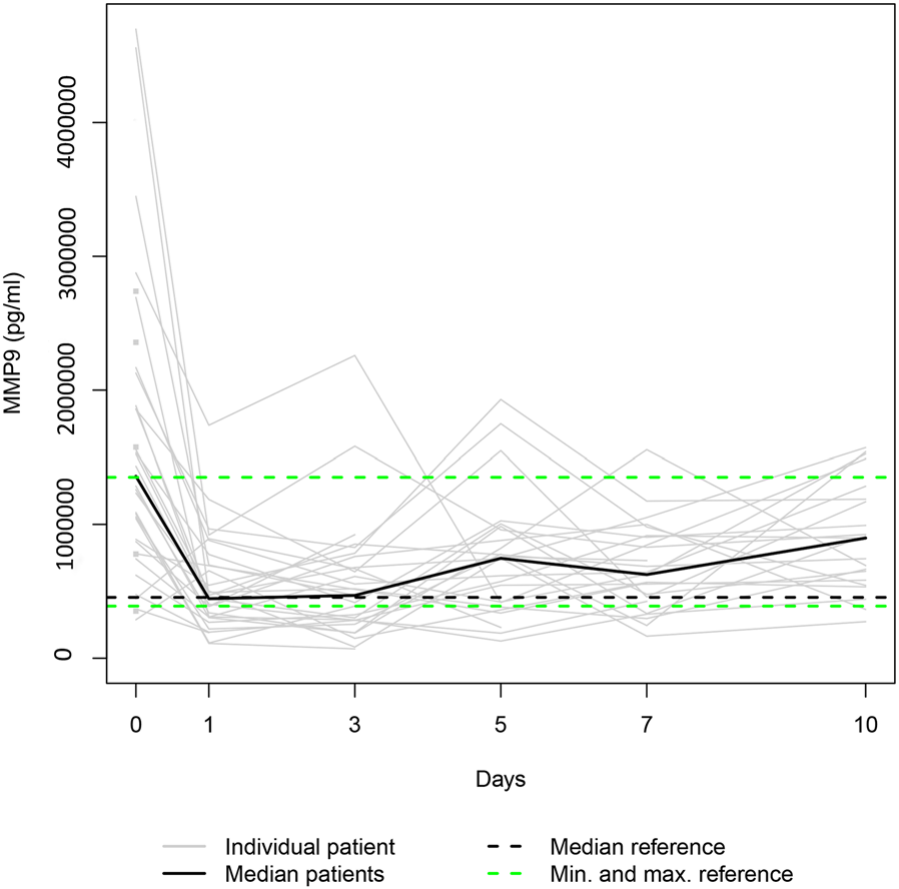
The study group’s individual MMP9 serum levels (gray lines) and the median MMP9 serum level (bold black line). Median (dashed bold black line) and minimum and maximum (dashed green lines) MMP9 serum levels in the healthy volunteers. The gray squares on the plots represent the patients with measurements only at admission

**Fig. 3:**
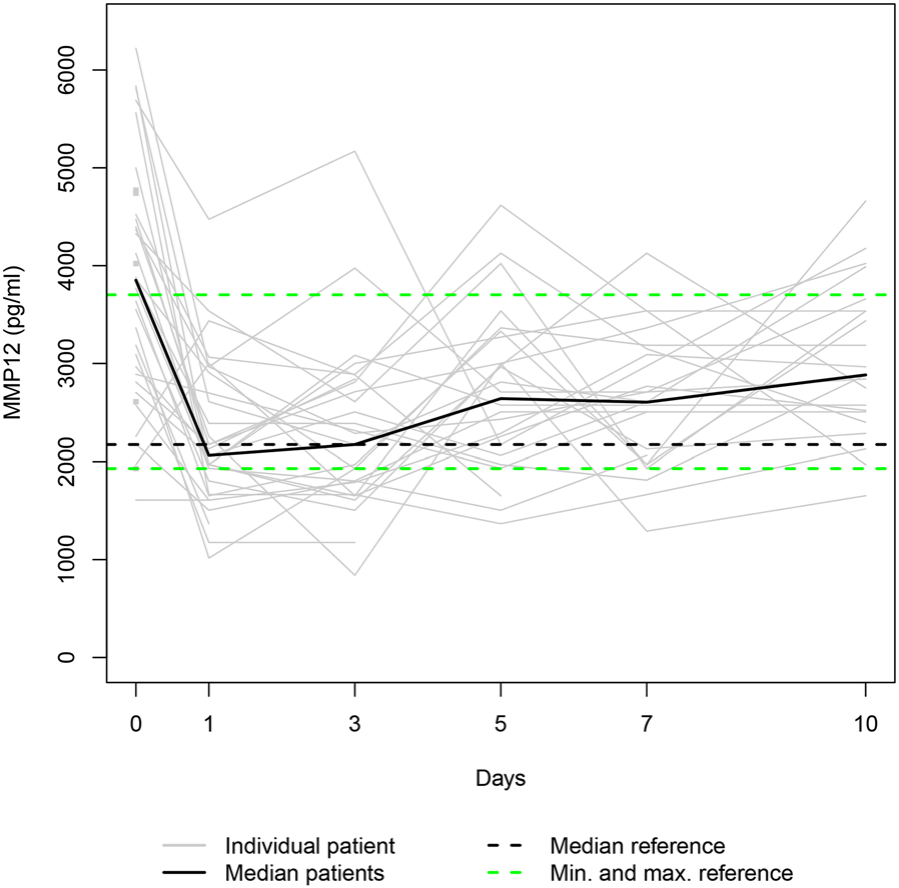
The study group’s individual MMP12 serum levels (gray lines) and the median MMP12 serum level (bold black line). Median (dashed bold black line) and minimum and maximum (dashed green lines) MMP12 serum levels in the healthy volunteers. The gray squares on the plots represent the patients with measurements only at admission

**Fig. 4:**
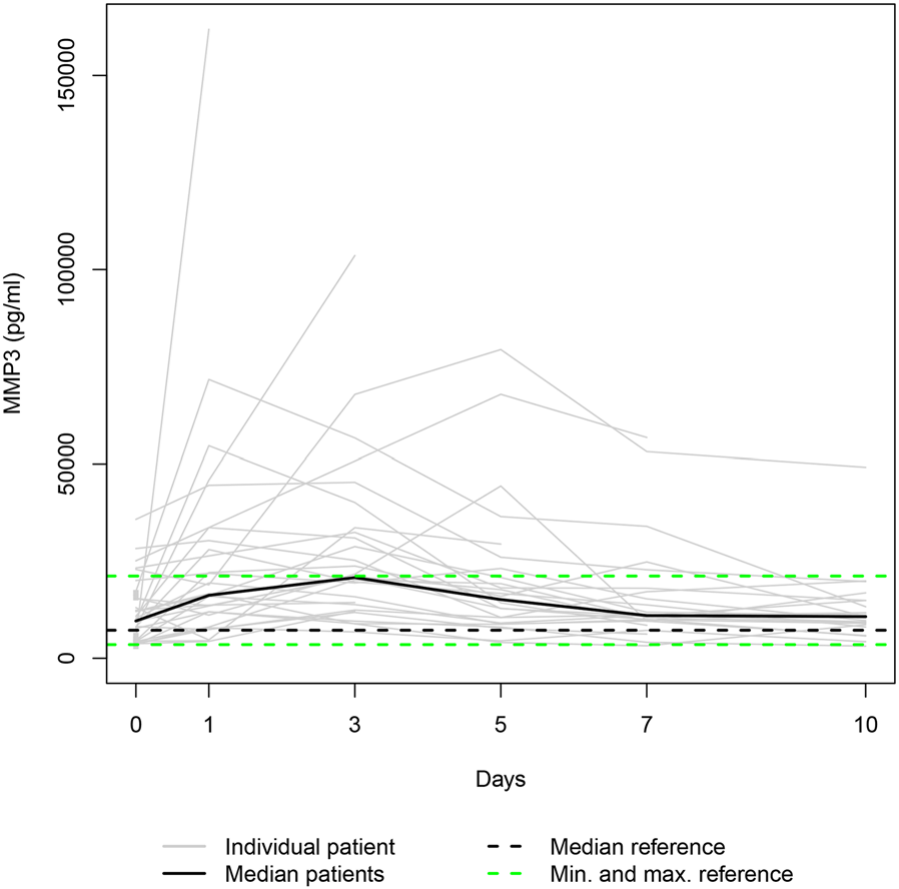
The study group's individual MMP3 serum levels (gray lines) and the median MMP3 serum level (bold black line). Median (dashed bold black line) and minimum and maximum (dashed green lines) MMP3 serum levels in the healthy volunteers. The gray squares on the plots represent the patients with measurements only at admission

**Fig. 5:**
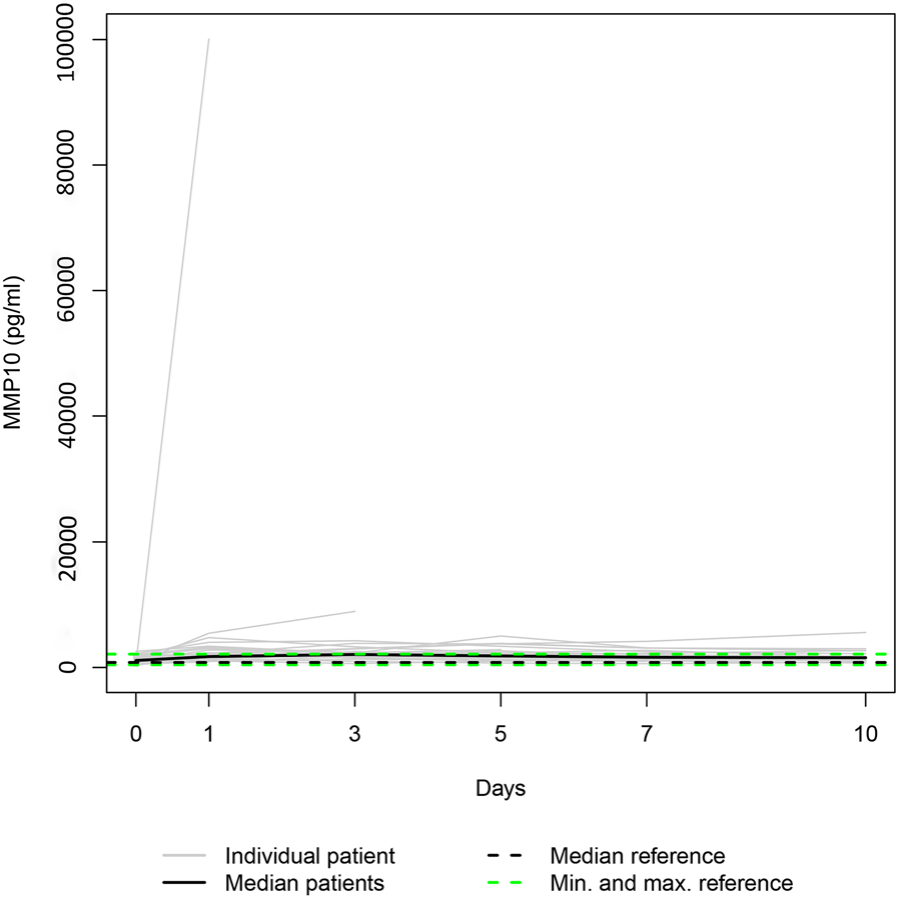
The study group's individual MMP10 serum levels (gray lines) and the median MMP10 serum level (bold black line). Median (dashed bold black line) and minimum and maximum (dashed green lines) MMP10 serum levels in the healthy volunteers. The gray squares on the plots represent the patients with measurements only at admission

**Fig. 6:**
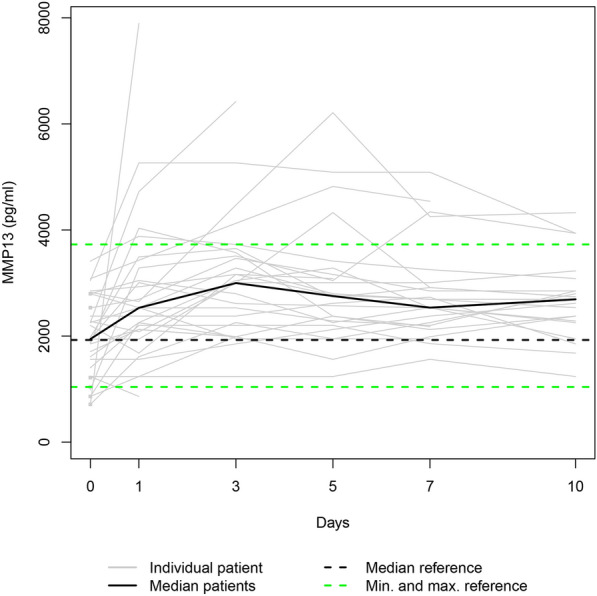
The study group’s individual MMP13 serum levels (gray lines) and the median MMP13 serum level (bold black line). Median (dashed bold black line) and minimum and maximum (dashed green lines) MMP13 serum levels in the healthy volunteers. The gray squares on the plots represent the patients with measurements only at admission

Due to the fatalities, our data set was only complete for day 0. Additionally, three patients refused consent for further blood draws when they attained consciousness on days 1, 5, and 7, respectively. Consequently, 36, 31, 28, 25, 24, and 23 samples were available for enzyme level assessment on days 0, 1, 3, 5, 7, and 10. Figures 1, 2, 3, 4, 5, and 6 display the trend curves for the individual serum levels of MMP3, MMP8, MMP9, MMP10, MMP12, and MMP13 and their medians.

Since the individual enzyme levels covered a wide range, we focussed on the trajectories of the medians on a smaller scale to show similarities in course progressions.

#### MMP8, MMP9, and MMP12

**Fig. 7:**
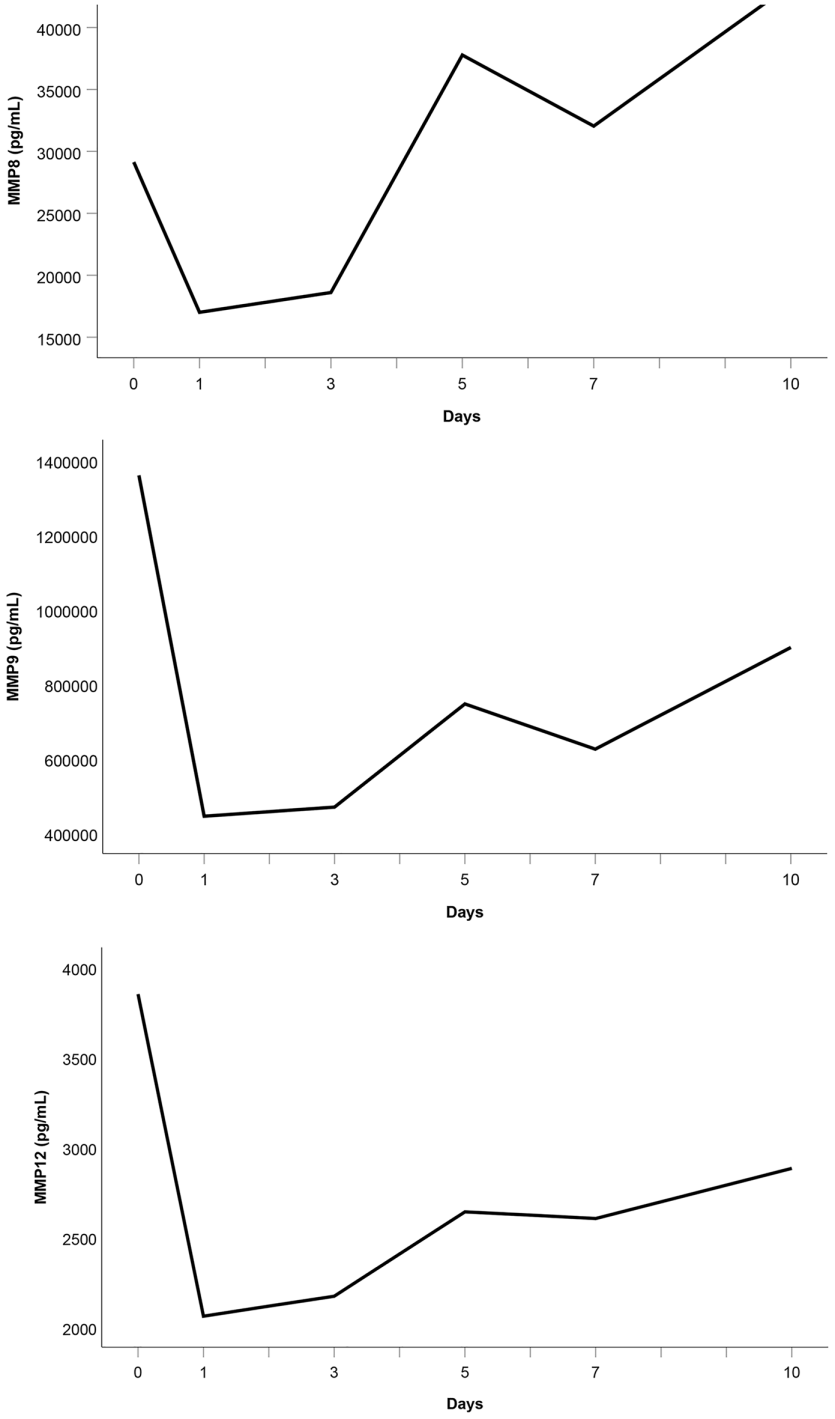
**a** Median MMP8 serum level, **b** median MMP9 serum level, and **c** median MMP12 serum level in the study group

As Fig. 7 suggests, the median serum levels of MMP8, MMP9, and MMP12 significantly decreased from their initial assessment at admission to day 1 (*p* ≤ 0.002), whereas they all increased from day 1 to day 10 (*p* ≤ 0.014).

The Spearman correlation coefficients were calculated to determine the inter-relationship of the MMP8, MMP9, and MMP12 levels at the same measurement points. They are presented in Table 2.

**Table 2:** Spearman correlations between enzyme serum levels

	**MMP8 day 0**	**MMP9 day 0**	**MMP12 day 0**
MMP8 day 0	1	0.895**	0.871**
MMP9 day 0	0.895**	1	0.982**
MMP12 day 0	0.871**	0.982**	1
	**MMP8 day 1**	**MMP9 day 1**	**MMP12 day 1**
MMP8 day 1	1	0.661**	0.649**
MMP9 day 1	0.661**	1	0.954**
MMP12 day 1	0.649**	0.954**	1
	**MMP8 day 3**	**MMP9 day 3**	**MMP12 day 3**
MMP8 day 3	1	0.878**	0.867**
MMP9 day 3	0.878**	1	0.951**
MMP12 day 3	0.867**	0.951**	1
	**MMP8 day 5**	**MMP9 day 5**	**MMP12 day 5**
MMP8 day 5	1	0.854**	0.874**
MMP9 day 5	0.854**	1	0.937**
MMP12 day 5	0.874**	0.937**	1
	**MMP8 day 7**	**MMP9 day 7**	**MMP12 day 7**
MMP8 day 7	1	0.639**	0.562**
MMP9 day 7	0.639**	1	0.960**
MMP12 day 7	0.562**	0.960**	1
	**MMP8 day 10**	**MMP9 day 10**	**MMP12 day 10**
MMP8 day 10	1	0.836**	0.807**
MMP9 day 10	0.836**	1	0.963**
MMP12 day 10	0.807**	0.963**	1

^**^The correlation is significant at a level of 0.01 (two-sided)

#### MMP3, MMP10, and MMP13

**Fig. 8:**
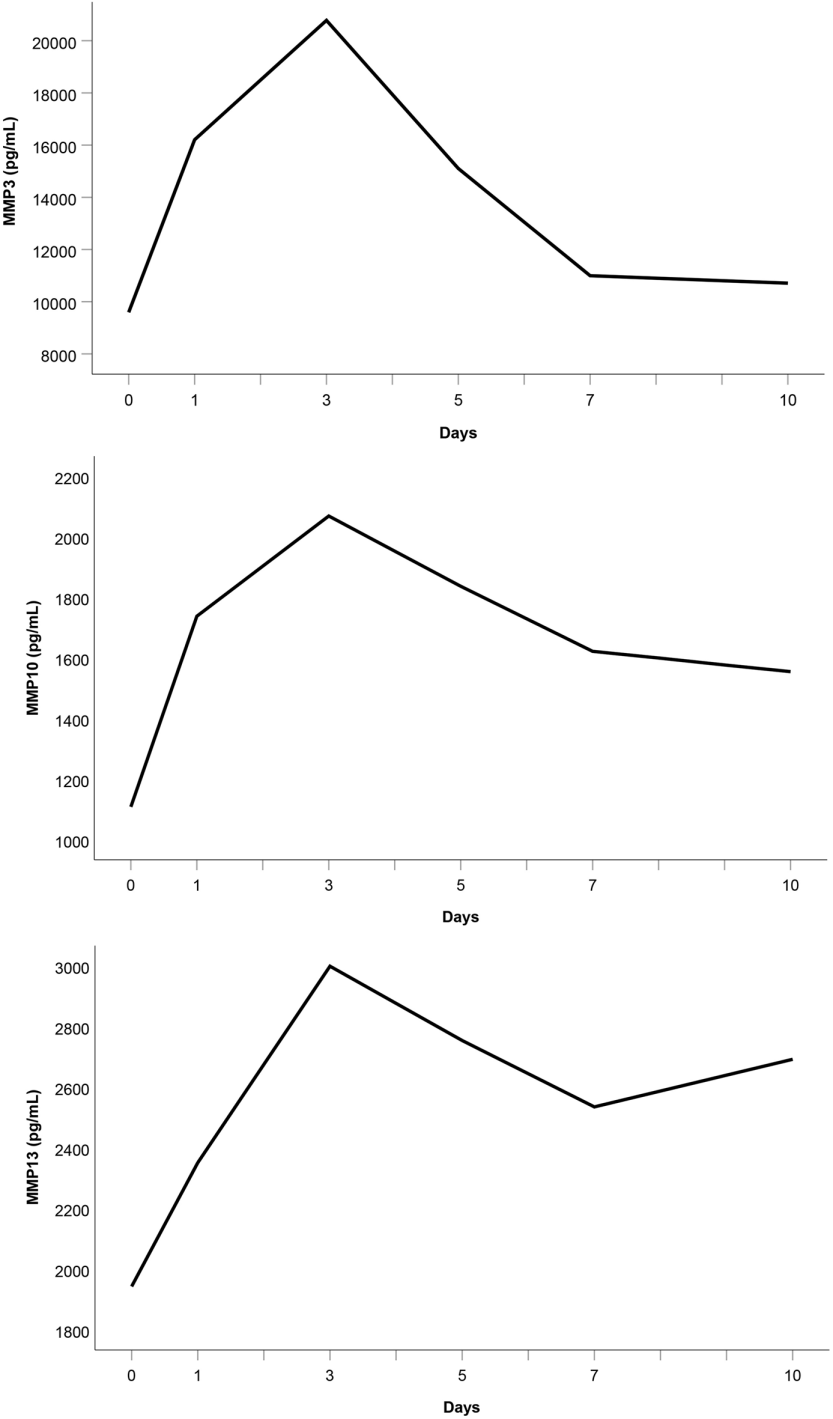
**a** Median MMP3 serum level, **b** median MMP10 serum level, and **c** median MMP13 serum level in the study group

As Fig. 8 indicates, the median serum levels of MMP3, MMP10, and MMP13 significantly increased from admission to day 3 (*p* < 0.001). However, only MMP8 and MMP9 serum levels significantly decreased from day 3 to day 10 (*p* ≤ 0.027).

The Spearman coefficients for the MMP3, MMP10, and MMP13 serum levels are displayed in Table 3.

**Table 3:** Spearman correlations between enzyme serum levels

	**MMP3 day 0**	**MMP10 day 0**	**MMP13 day 0**
MMP3 day 0	1	0.663**	0.895**
MMP10 day 0	0.663**	1	0.564**
MMP13 day 0	0.895**	0.564**	1
	**MMP3 day 1**	**MMP10 day 1**	**MMP13 day 1**
MMP3 day 1	1	0.747**	0.967**
MMP10 day 1	0.747**	1	0.741**
MMP13 day 1	0.967**	0.741**	1
	**MMP3 day 3**	**MMP10 day 3**	**MMP13 day 3**
MMP3 day 3	1	0.536**	0.937**
MMP10 day 3	0.536**	1	0.512**
MMP13 day 3	0.937**	0.512**	1
	**MMP3 day 5**	**MMP10 day 5**	**MMP13 day 5**
MMP3 day 5	1	0.717**	0.911**
MMP10 day 5	0.717**	1	0.687**
MMP13 day 5	0.911**	0.687**	1
	**MMP3 day 7**	**MMP10 day 7**	**MMP13 day 7**
MMP3 day 7	1	0.474*	0.769**
MMP10 day 7	0.474*	1	0.599**
MMP13 day 7	0.769**	0.599**	1
	**MMP3 day 10**	**MMP10 day 10**	**MMP13 day 10**
MMP3 day 10	1	0.547*	0.614**
MMP10 day 10	0.547*	1	0.366
MMP13 day 10	0.614**	0.366	1

^*^The correlation is significant at a level of 0.05 (both sides)

^**^The correlation is significant at a level of 0.01 (both sides)

### Auto- and cross-correlations of enzyme-level-time-series with a lag of 2 days

To assess relations between enzyme levels measured with a time difference of 2 days, we calculated the auto- and the cross-correlations between the time series of days 1, 3, and 5 and the time series of days 3, 5, and 7 of all possible pairs of measured enzymes. The relevant coefficients are presented in Table 4. With data from 22 to 36 patients, the correlation analysis had a power of 0.67 to 0.88 when the true correlation was 0.5.

**Table 4:**
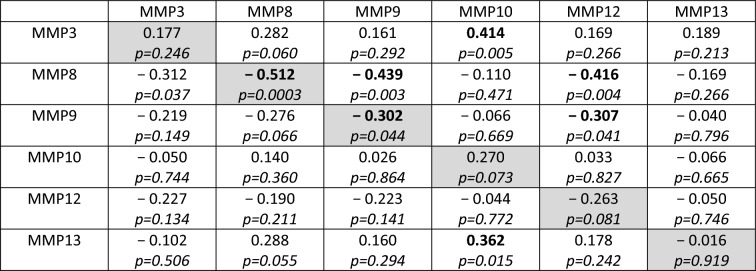
Auto- and cross-correlation matrix

Autocorrelations are presented in the primary diagonal (gray cells). The autocorrelation represents the correlation of each variable on days 1–5 with later measurements of the same variable (days 3–7). Off-diagonal entries represent the average within-subject correlation between two variables measured on the same day

### Univariable logistic analyses

One-third of the fatalities had already died on day 1 after polytrauma. Therefore, we conducted univariable logistic regression analyses to answer whether enzyme levels could predict in-hospital mortality with the day 0 level of each selected enzyme as the independent variable. Since they had a wide range, we divided them by 1000 as the first step, meaning each OR refers to a difference of 1000 units (pg/mL). The results of the univariable logistic regression analyses are presented in Table 5, solely identifying the MMP10 day 0 level as a significant predictor of in-hospital mortality.

**Table 5:** Univariable logistic regression of every enzyme level at admission

Predictor	OR	95% CI		*p-* value
		Lower	Upper	
MMP3 day 0 × 10^−3^	0.899	0.794	1.017	0.091
MMP8 day 0 × 10^−3^	0.998	0.990	1.006	0.592
MMP9 day 0 × 10^−3^	1.000	0.999	1.001	0.877
MMP10 day 0 × 10^−3^	0.187	0.036	0.978	0.047
MMP12 day 0 × 10^−3^	0.876	0.502	1.529	0.642
MMP13 day 0 × 10^−3^	0.445	0.167	1.185	0.105

To further aid in interpreting the result—the MMP10 day 0 level ranged from 392 to 2570 pg/mL—we also calculated the OR for a difference of 200 units and obtained 0.715 (95% CI 0.514 − 0.996; *p* = 0.047).

### ROC statistics

**Fig. 9:**
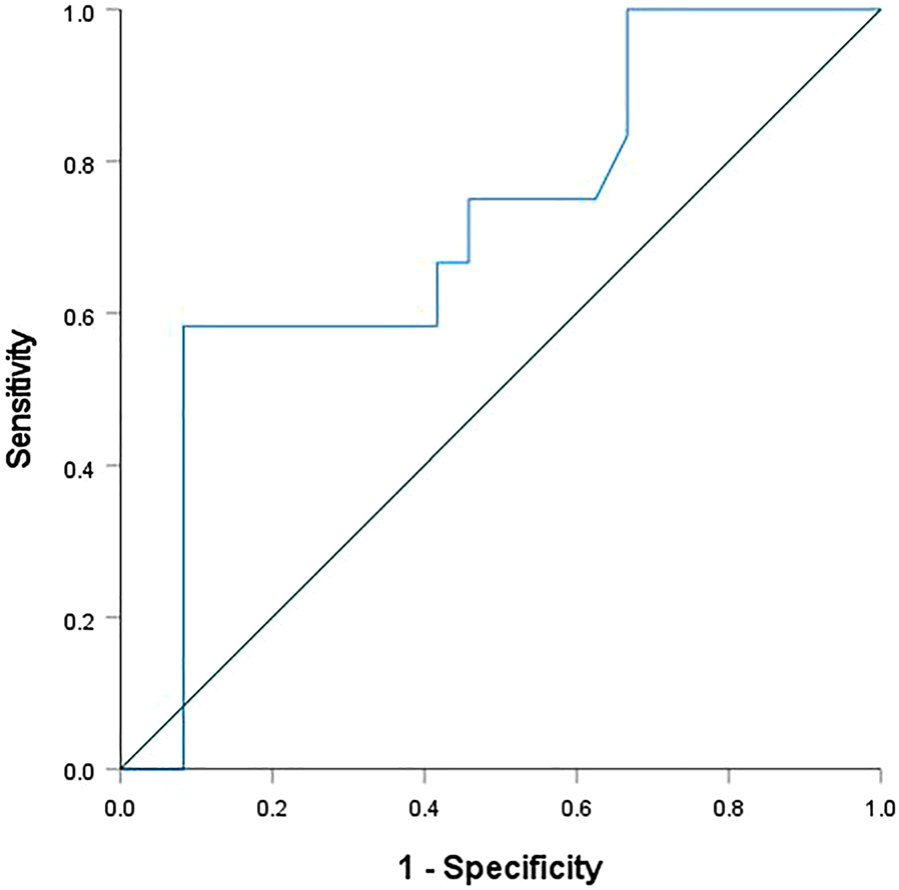
ROC curve for MMP10 serum levels and in-hospital mortality assessed at admission

We plotted the ROC curve for the MMP10 day 0 level for graphical analysis (Fig. 9), determining a cutoff level of 1256 pg/mL (sensitivity, 0.750; specificity, 0.542) and an AUC = 0.714. This cutoff level correctly identifies nine out of the 12 fatalities and misclassifies 11 out of the 24 survivors.

### Boxplots

**Fig. 10:**
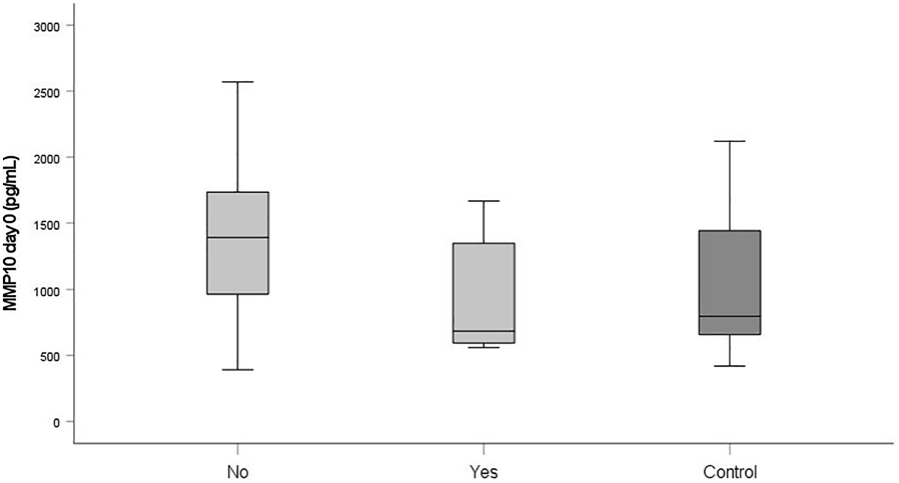
Boxplots displaying the MMP10 serum levels of the fatalities (Yes) and the survivors (No) assessed in the study group at admission and in the healthy volunteers

At admission, median MMP10 levels were significantly lower in fatalities than in survivors (685 [559–667] pg/mL versus 1359 [392–570] pg/mL, *p* = 0.038). The relevant boxplots and the boxplot of the control group are displayed in Fig. 10.

## Discussion

Our data demonstrate either an immediate or delayed up-regulation of MMP serum levels after the polytrauma, in each case indicating their crucial role in the endogenous defense response to severe traumatic injury. Time trajectories were similar between MMP8, MMP9, and MMP12, and between MMP3, MMP10, and MMP13, respectively. For a two-day lag, we calculated a significant autocorrelation for MMP8 and MMP9 and significant cross-correlations between MMP8 and MMP9, MMP8 and MMP12, MM9 and MMP12, MMP3 and MMP10, and MMP13 and MMP10. Finally, the MMP10 serum level assessed at admission was associated with in-hospital mortality in polytraumatized patients.

MMPs are essential to the network of multidirectional communication within tissues and cells, regulating cell proliferation and differentiation, tissue homeostasis, and innate and adaptive immune response [33]. Their release into circulation after polytrauma results in individual trend lines of the patients that show strongly varying time courses (Figs. 1–6). Focussing on the trajectories of the medians, we revealed similar course progressions for MMP8, MMP9, and MMP12 levels on a highly different size scale. They increased sharply from the time of injury until the hospital admission, dropped within the next day, and—while going through fluctuations—finally rose until day 10 (*p* ≤ 0.014). Additionally, Table 2 reveals there is, in most cases, a very strong association between each two of the three enzyme levels assessed at the same measurement point. Thus, we hypothesize that the triggers for their release (injury mechanism, location, and extent; damaged cell types) were similar. We also revealed similar time progressions for the MMP3, MMP10, and MMP13 levels. However, for this group, an (almost) strong association between every 2 levels existed only until day 7.

We used within-patient cross-correlations to determine if two enzymes are “causative” related. Since there is a time delay between impact and effect, we computed the coefficients of repeated measurements between the time series of one enzyme level (assessed on days 1, 3, and 5) and the 2-day-lagged version of the time series of another enzyme level (evaluated on days 3, 5, and 7). In Table 4, significant negative cross-correlation coefficients are presented between MMP8 and MMP9 (− 0.439), MMP8 and MMP12 (− 0.416), and MMP9 and MMP12 levels (− 0.307). This fact indicates that a rise in the first-mentioned enzyme level in a patient contributes to a fall in the second-mentioned one 2 days later. Contrarily, the significant positive cross-correlation coefficients 0.414 and 0.362 reveal that an increase in a patient’s MMP3 or MMP13 level co-causes an increase in the MMP10 level 2 days later.

In our study, autocorrelation refers to the repeated measurement correlation of a time series with its 2 day-lagged version. Table 4 presents significant autocorrelation coefficients only for MMP8 (− 0.512) and MMP9 (− 0.302). The negative values indicate that an increase observed in a time interval leads to a proportionate decrease in the lagged time interval. Negative autocorrelation becomes apparent in large fluctuations as it is noticeable in the time trajectories of the median enzyme levels of MMP8 and MMP9 (Fig. 7).

Univariable binary regression analysis revealed a significant relationship between the MMP10 level assessed at admission and in-hospital mortality. We calculated an OR of 0.715 for a difference of 200 units, indicating that any increase of the MMP10 level by 200 pg/mL decreases the odds of dying by 28.5%. ROC curve analysis provided a 1256 pg/mL cutoff for the MMP10 day 0 level and in-hospital mortality. According to the sensitivity of 0.750 and the specificity of 0.542, the true positive rate using this cutoff level is 75%, and the true negative rate is 45.8%. Calculating the AUC is an effective way to summarize its overall diagnostic accuracy. Unfortunately, 0.714 is considered not clinically useful in general [34]. Therefore, if at all, MMP10 is only suitable as a constituent of a biomarker panel that covers multiple pathways to identify polytraumatized patients with a high risk of dying.

Notably, the range of the MMP10 levels of the control group encompassed the MMP10 day 0 levels of all fatalities (Fig. 10). Since the OR of 0.715 indicates a protective effect in polytraumatized patients, administering MMP10 at hospital admission might reduce their risk of dying. In general, MMPs 3, 8, 9, 10, 12, and 13 serum levels that strongly deviate from the norm, as presented in Figs. 1−6, or an imbalance between the MMP level and its natural inhibitor levels, might impair the healing process of the injuries. Regulating the enzyme concentration in circulation during hospitalization might optimize the endogenous defense response after polytrauma, reducing mortality and morbidity rates.

The heterogeneity of polytrauma victims, resulting from myriad etiologies and injury combinations, makes it challenging to identify clinically relevant biomarker candidates that are significant in any situation, regardless of the concurrent injuries and the selected inclusion and exclusion criteria. In our opinion, there is no single biomarker that can accurately predict complications and mortality in polytraumatized patients upon hospital admission. Therefore, we intend to conduct further pilot studies to evaluate the serum levels of various protein families. Hopefully, each study will contribute a small piece to a biomarker panel. Once we finished the puzzle, we plan to conduct a large prospective study at our Level I trauma center to assess the serum levels of the selected biomarker candidates over time, paying close attention to their clinical relevance when working together.

The limitations of our study include the fact that we based our sample size on the number of patients reported in published pilot studies [2, 35–39] and did not perform an a priori power analysis. Furthermore, blood sampling was determined by the patient's willingness, thus resulting in an incomplete data set of enzyme levels for three survivors. Finally, the study population was recruited only in one trauma center.

## Conclusions

We relied on standard methods to show similarities in the selected MMP levels’ course progression and determine their ability to predict mortality. Contrarily, we used an innovative approach to reveal whether a change in a specific serum MMP level has a delayed impact on the serum level of the same or of another MMP in an individual. We proved a lead–lag effect for several MMP pairs, indicating that the associated enzymes interact serially. Hopefully, our results will boost further investigation by basic studies to provide underlying causes for our findings. At best, they might lead to scenarios for regulating MMPs’ activity, offering an attractive therapeutic perspective in polytrauma management.

## Data Availability

The datasets used and analyzed during the current study are available from the corresponding author upon reasonable request.
